# Islands of cooperation emerge by stigmergic interactions in iterated spatial games

**DOI:** 10.1371/journal.pone.0306915

**Published:** 2024-07-12

**Authors:** Franco Zambonelli, Federico Bergenti, Stefano Mariani, Stefania Monica

**Affiliations:** 1 Dipartimento di Scienze e Metodi dell’Ingegneria, Università di Modena e Reggio Emilia, Reggio Emilia, Italy; 2 Istituto di Informatica e Telematica (IIT), Consiglio Nazionale delle Ricerca, Pisa, Italy; 3 Dipartimento di Scienze Matematiche Fisiche e Informatiche, Università di Parma, Parma, Italy; University of Electronic Science and Technology of China, CHINA

## Abstract

This article focuses on the emergence of cooperation in societies of self-interested agents. In particular, it introduces a mechanism based on indirect—stigmergic—interactions between agents moving in an environment, to express the likeliness of finding cooperative partners. On the one hand, agents that find themselves cooperating with others emit pheromones in their current location, expressing the presence of agents willing to cooperate. On the other hand, agents that sense pheromones tend to move towards regions with a higher pheromone concentration. Results show that this mechanism leads to the emergence of spatial regions where cooperation can be effectively sustained, and in which agents can overall get better payoffs than those agents not taking into account pheromones in their choices.

## Introduction

Cooperation tends to emerge in real-world societies of self-interested agents, as it has been observed at the level of biological processes, multicellular organisms, and even of human societies [1, 2]. Yet, such phenomenon is puzzling: when helping others has a price for the helpers—as it is in most cases—why should cooperation emerge and sustain?

Exemplifying this as a multiagent game [3], specifically the Prisoner Dilemma (PD), one can consider two agents that meet with each other and that simultaneously have to choose one of the two strategies Cooperation (C) or Defection (D). According to the game, each agent receives a benefit based on the strategies adopted by the two: if both agents choose cooperation, both will obtain a reward *R*; if one chooses cooperation and the other one chooses defection, the former will obtain a payoff *S* < *R* but the latter will get a payoff *T* > *R*; if both choose to defect, they will get a payoff *P* such that *S* < *P* < *R*. On this basis, the rational choice for an agent during the encounter is necessarily defection, and no chance for the emergence of sustainable cooperative behavior seems to exist—in fact, defection for both agents is the game unique Nash equilibrium [4].

However, since cooperative behaviors are indeed exhibited by many real-world systems that can be modelled as the PD, a variety of mechanisms have been hypothesized and studied that can lead to the emergence of cooperation, despite the rational tendency of individual agents towards defection [5]: direct reciprocity [6], indirect reciprocity and reputation-based mechanisms [7–9], and agent migration [10].

In this article, we aim to experiment with a mechanism based on stigmergic—pheromones-based—interactions between agents, as those found in ant colonies and other classes of organisms [11]. Agents moving in a spatial environment that find other cooperating agents in their current location emit pheromones, expressing the presence in that location of agents willing to cooperate. So, pheromones represent a sort of external, spatially situated, reputation memory, expressing the areas of the environment in which cooperation can be found.

We have experimented our approach in an extended spatial version of Axelrod PD tournament [1]. Our results show that:

- Stigmergic interactions lead to the emergence of spatial “islands of cooperation”, where cooperative behavior can be effectively sustained;
- Agents relying on pheromones for their choices can overall get higher payoffs than agents following different—non pheromone-based—strategies.

## Related work

Over the years, many mechanisms have been hypothesized and studied that can lead to the emergence of cooperation in societies of self-interested agents [5].

Direct reciprocity [6] makes one agent strategically akin to cooperate with those agents that cooperated with it in the past. However, such mechanisms tend to promote the emergence of local—typically segregated—communities. Indeed, direct reciprocity and even forced segregation mechanisms [12] can promote cooperation but only at the small scale. Our approach, on the other hand, promotes the emergence of large groups of non segregated cooperators.

Indirect reciprocity [7] makes one agent akin to cooperate with those agents that cooperated with others in the past. This somehow requires agents to have access to information related to the past actions of other agents. For instance, a value can be associated to each agent, expressing how much such agent has cooperated with other agents in the past. In other words, a public reputation or trust value is associated with each agent [8, 9]. The pheromones adopted in our approach can be somehow considered a form of reputation, although not associated with individual agents but externalized and associated with regions of the environment.

A reputation-based mechanism to promote the emergence of cooperation in systems of mobile agents, somewhat resembling ours, is described in [13]. There, each agent holds a reputation value expressing its past willingness to cooperate, and this value can be perceived by agents in the neighbourhood. On this base, as agents move in the environment, they perceive the reputation of the agents in the neighbourhood and they move with higher probability in the direction where the overall reputation is higher. Our approach generalizes this one, by having pheromones acting as a sort of externalized distributed reputation field associated with regions of the environment, thus eventually supporting more stable regions of cooperation.

Strictly related to the above, recent studies have shown that imitation-based mobility, i.e., having agents preferentially move towards those directions in which a large number of agents have already moved, can lead to the emergence of spatial groups of cooperating agents [10]. Our approach promotes migration towards groups of cooperating agents, but by exploiting the historical spatial information about cooperation embedded in pheromone concentration.

The evolutionary formation of spatial patterns of cooperation in the presence of mobile agents is discussed in [14]. In particular, it is shown that regions of cooperation in a spatial environment (with patterns similar to the ones we have observed in our experiments) can appear by increasing the reproduction rate of cooperator agents in a region proportionally to the density of cooperators in that region. To some extent, our approach adopts pheromones as a mean to express the density of cooperators in a region, and to attract existing cooperators (rather than generating new ones) in that region. Spatial patterns of cooperation are also studied in [15], but for static agents. Authors study the emergence of such patterns in different network topologies and especially under a mechanism of direct reciprocity called “conditional punishment“. Namely, defective behaviour is sanctioned proportionally to the number of other punishing agents—instead of unconditionally. Our approach shares the interest in spatial cooperation, but studies mobile agents instead, and the role of pheromones as externalised memory. In [16] there is another detailed investigation of emergence of cooperative behaviours with a focus on network topology, but in the framework of evolutionary games. There, both the game played by agents and their strategies in playing such games can vary in time. The authors study thus the conditions upon which cooperation emerges despite such dynamism.

In [17] it is shown that, in order to promote cooperation beyond small groups, it is necessary to promote inter-group interactions, so as to somewhat diffuse cooperative behaviors. Our approach promotes such types of inter-group interactions, in that pheromones diffuse in the environment and this can attract passing-by agents in a cooperative island.

Another mechanism that has been explored to promote the emergence of cooperation is partner selection [18]. There, agents are informed about the most recent past actions of the partner they encounter, and can decide not to engage in the game with partners having a history of defections. In our approach, we do not explicitly enforce partner selection, although the migration of agents in regions of high cooperation is an implicit form of partner selection. A somewhat related mechanism is proposed in [19], where an agent chooses its strategy based on the past payoffs of its partners. That is, by copying the strategy of its neighbors having accumulated higher payoffs in the past.

The approach we propose here differs from all the above because it summarises in a single mechanism and externalises to the shared environment inhabited by agents all the metadata necessary to improve cooperation (be it a form of reputation or past interaction histories). Thus, our proposal reduces complexity while enabling effective cooperative behaviours, as shown experimentally.

## The stigmergic approach to cooperation

In this section, after introducing the notion of stigmergy and pheromone-based communication, we describe and formalize our proposed strategy to foster emergence of islands of cooperation in a population of selfish agents.

### Background

Stigmergy, that is the exploitation of signals left in the environment to somewhat indirectly communicate with others and/or affect their behaviors [20], is a mechanisms widely exploited in natural as well as in artificial (ICT) systems as a mean to promote coordinated behaviors.

For instance, in nature, many species—such as ants and slime molds—exploit indirect stigmergic interactions in the form of pheromones [21] or other types of chemicals [22]. This enables colonies of such species to achieve collective goals in a coordinated and cooperative way without any centralised supervision or control. For instance, ants deposit pheromones to signal that they have found food, thus leading to the creation of pheromone trails in the environment. Such trails help all the members of the colony in finding food efficiently. Consequently, inspired by such natural phenomena, many approaches to coordination in distributed computing systems have been proposed that rely on similar stigmergic means of interactions, typically in the forms of digital pheromones [23] or virtual gradient fields [24], to support coordination problems such as message routing and coordinated spatial movements in robot teams [25].

In the above example, the emission of pheromones (whether natural or digital) can be considered as a way to externalize information about the status of the individuals (e.g., “I have food!”) and/or of the environment (e.g., “there is food here!”) and create a shared collective memory about the overall status of the system. However, in several cases, pheromones can be interpreted also as a mean to externalize emotions, whether positive or negative ones, by the individuals of a system (e.g., “I am happy I have found food here!”). The result is, eventually, in the global perception of a collective emotional status that implicitly leads to cooperation. Indeed, in psychology, it is known that signals of emotion can effectively promote cooperation in humans [26].

A further alternative way to interpret pheromones is as a sort of “reputation” of the environment [13]. Leaving a pheromone in a region of the environment can tell the members of the system that the region has some specific good (or bad) properties, and thus that it is worth to approach (or stay away from) that region by following uphill (or downhill) the gradient of the pheromones intensity. With regard to the last point, it is worth emphasizing that in stigmergic interactions a primary role is played by the mechanisms of pheromone diffusion and evaporation:

- When a pheromone is deposited in a point of the environment, it gradually spatially diffuses around. This induces the creation of a gradient field with a peak of intensity at the original deposit point.
- Pheromones gradually evaporate, and if not reinforced with further deposit they gradually disappear. This ensures that when the reputation property does not longer apply to a region of space, the associated pheromone trace will accordingly disappear, eventually.

### The stigmergic strategy

Based on all the above considerations, we have thought at exploiting stigmergy to let agents roam in an environment to signal whether in some regions of the environment they have encountered cooperators, and to let them follow pheromone trails in order to find cooperators.

In short, the implemented strategy, which we simply call the “Stigmergic” strategy, works as follows:

- When agents do not “smell” any pheromone scent in their current location, they keep on walking randomly in the environment;
- When meeting another agent in the same location, they select randomly whether to cooperate or defect;
- After an encounter, if the partner cooperated the agent emits the pheromone
- Whenever an agent perceives pheromones in the environment, it preferentially walks in the direction of growing pheromone intensity;
- When the pheromone intensity perceived by an agent is over a specified threshold *T*_*i*_, it starts cooperating with the other agents it meets.

The specific type of random walk adopted by agents is the so called *correlated random walk* [27]: the direction followed by the agent at each step is—unlike in Brownian motion—correlated with the direction of the previous step. Specifically, in our implementation, the direction *d* is computed starting from the direction at the previous step, modified of an angle (called *wiggle angle*) at random, in any case included between $-\frac{\pi }{2}$ and $\frac{\pi }{2}$ to ensure correlation with the previous direction.

Instead, in the presence of pheromones, agents switch behavior to the so called *biased random walk* [27, 28]: the direction chosen at each time step is biased toward that of the growing pheromone intensity (in other terms, agents movements are driven by chemotaxis [29]). Specifically, when the agents sense pheromones ahead (i.e., within a cone of visibility centered in the direction of movement and spanning *sniff angle* degrees on each side), the direction is computed starting from that of higher pheromone intensity, again modified of the random wiggle angle to ensure that agents can also from time to time “escape” from those regions of high pheromone intensity.

### Formalization of the stigmergic strategy

The proposed strategy can be studied using the following formalization. Without loss of generality, an environment $E=\left[0,b\right)\times \left[0,h\right)\subset R^{2}$, with $b\in N_{+}$ and $h\in N_{+}$, is considered. The environment is split into *bh* disjoint patches. Each patch is a square whose base and height equal 1. The patches are uniquely identified via the position of their lower-left corners, and therefore the patch (*i*, *j*), where 0 ≤ *i* < *b* and 0 ≤ *j* < *h*, is the square $\left[i,i+1\right)\times \left[j,j+1\right)\subset R^{2}$. Given a point **x** ∈ *E*, patch(x) is the unique patch that contains **x**.

Each patch has a neighborhood of 8 patches using a toroidal topology. Therefore, given a patch (*i*, *j*), its neighborhood *N*(*i*, *j*) is the set formed by the eight patches (*i*, (*j* ± 1) ⊘ *h*), ((*i* ± 1) ⊘ *b*, *j*), ((*i* ± 1) ⊘ *b*, (*j* ± 1) ⊘ *h*), where *p* ⊘ *q* is the remainder of the division between $p\in R_{+}$ and $q\in R_{+}$. Given two points **u** ∈ *E*, with **u** = (*u*_1_, *u*_2_), and **v** ∈ *E*, with **v** = (*v*_1_, *v*_2_), **u** ⊕ **v** = ((*u*_1_ + *v*_1_) ⊘ *b*, (*u*_2_ + *v*_2_) ⊘ *h*). Given a point **u** ∈ *E* and a direction identified by an angle *α* ∈ [−*π*, *π*) (as usual, with respect to the x-axis and positive counter-clockwise), ahead(u,α,l) is the single patch that intersects at distance $l\in R_{+}$ the half line from **u** in the direction *α*.

The considered world includes n∈N agents that are uniquely identified by a natural number from 1 to *n*. The agents move in the environment at discrete time instants, and given an agent *a*, its position at time t∈N is denoted by **x**_*a*_(*t*) ∈ *E* and its heading direction is denoted by *α*_*a*_(*t*) ∈ [−*π*, *π*). The agents iteratively play rounds of the considered game and, given an agent *a*, *s*_*a*_(*t*) ∈ {*C*, *D*, *N*} is the action chosen for the round at time *t*, where the label *N* is used to indicate that the agent did not play a round of the game at time *t*. If *s*_*a*_(*t*)≠*N*, then oppnt_*a*_(*t*) evaluates to the unique identifier of the agent that played against *a* at time *t*.

Given a patch (*i*, *j*), *ϕ*(*i*, *j*, *t*) is the amount of pheromones in the patch at time t∈N and *ρ*(*i*, *j*, *t*) is the number of agents whose positions are in the patch at time *t*. At time *t* = 0, the environment contains no pheromones and the agents are distributed randomly in the environment heading at random directions. Note that the following equation holds:

$$\begin{array}{c} \phi \left(i,j,t\right)=\sum_{a=1}^{n}{\text{at}}_{a}\left(i,j,t\right), \end{array}$$ (1)

where

$$\begin{array}{c} {\text{at}}_{a}\left(i,j,t\right)=\{\begin{array}{cc} 1 & \text{if}\;\text{patch}(x_{a}\left(t\right))=\left(i,j\right)\\ 0 & \text{otherwise} \end{array} \end{array}$$ (2)

The following equation formalizes the movements of an agent 1 ≤ *a* ≤ *n*:

$$\begin{array}{c} x_{a}\left(t+1\right)=x_{a}\left(t\right)⊕R\left(\alpha_{a}\left(t\right),{\tilde{\nabla }}_{a}\left(t\right),\theta \right)u, \end{array}$$ (3)

where **u** = (1, 0) and $R(\alpha_{a}\left(t\right),{\tilde{\nabla }}_{a}\left(t\right),\theta )$ is the clockwise rotation matrix obtained by summing the following angles:

- The current heading angle of agent *a*, namely *α*_*a*_(*t*);
- A random angle $\theta \in [-\bar{\theta },\bar{\theta }]$, where $\bar{\theta }\in \left[0,\frac{\pi }{2}\right]$ is the wiggle angle.
- An angle ${\tilde{\nabla }}_{a}\left(t\right)$ that account for the possible presence of pheromones and biases of the movement towards them.

In particular, in the absence of pheromones, ${\tilde{\nabla }}_{a}\left(t\right)=0$, expressing a correlated random walk. Otherwise, if the agent senses pheromones, we have:

$$\begin{array}{c} {\tilde{\nabla }}_{a}\left(t\right)=\underset{\gamma \in [\bar{\gamma },\bar{\gamma }]}{\text{argmax}\;}\phi \left(u,v,t\right)\;\text{and}\;\left(u,v\right)=\text{ahead}\left(x_{a}\left(t\right),\alpha_{a}\left(t\right)+\gamma ,1\right), \end{array}$$ (4)

where $\bar{\gamma }\in \left[0,\pi \right)$ is the *sniff angle* defining the cone of visibility, making an agent point towards one of the patches ahead with higher pheromone concentration to express a biased random walk.

Given a patch (*i*, *j*), the following equation formalizes how the pheromone concentration changes over time because of three phenomena, namely evaporation, diffusion, and agent emissions:

$$\begin{array}{c} \phi \left(i,j,t+1\right)=\phi \left(i,j,t\right)+\Delta_{e}\left(i,j,t\right)+\Delta_{d}\left(i,j,t\right)+\Delta_{p}\left(i,j,t\right). \end{array}$$ (5)

Evaporation contributes to the variation of the pheromones concentration as follows:

$$\begin{array}{c} \Delta_{e}\left(i,j,t\right)=-\epsilon \phi \left(i,j,t\right), \end{array}$$ (6)

where 0 < *ϵ* < 1 is a constant related to the evaporation rate. The diffusion contributes to the variation of the pheromone concentration as follows:

$$\begin{array}{c} \Delta_{d}\left(i,j,t\right)=\frac{\delta }{8}\sum_{\left(u,v)\in N(i,j\right)}\phi \left(u,v,t\right)-\delta \phi \left(i,j,t\right), \end{array}$$ (7)

where 0 < *δ* < 1 is a constant related to the diffusion rate. Finally, the contribution of the agents to the variation of the pheromone concentration is expressed as follows:

$$\begin{array}{c} \Delta_{p}\left(i,j,t\right)=\sigma \sum_{a\in C(t)}{\text{at}}_{a}\left(i,j,t\right) \end{array}$$ (8)

where $\sigma \in R_{+}$ quantifies the pheromone emission and

$$\begin{array}{c} C\left(t\right)=\{1\leq a\leq n\;|\;s_{a}\left(t\right)\neq N\wedge {\text{oppnt}}_{a}\left(t\right)=b\wedge s_{b}\left(t\right)=C\} \end{array}$$ (9)

represents the set of agents that had cooperative opponents at time *t*.

## Experiments

In this section we describe the experimental setting set up for assessing the performance of our proposed strategy, and report both qualitative and quantitative results stemming from experiments.

### Setup

**Fig 1:**
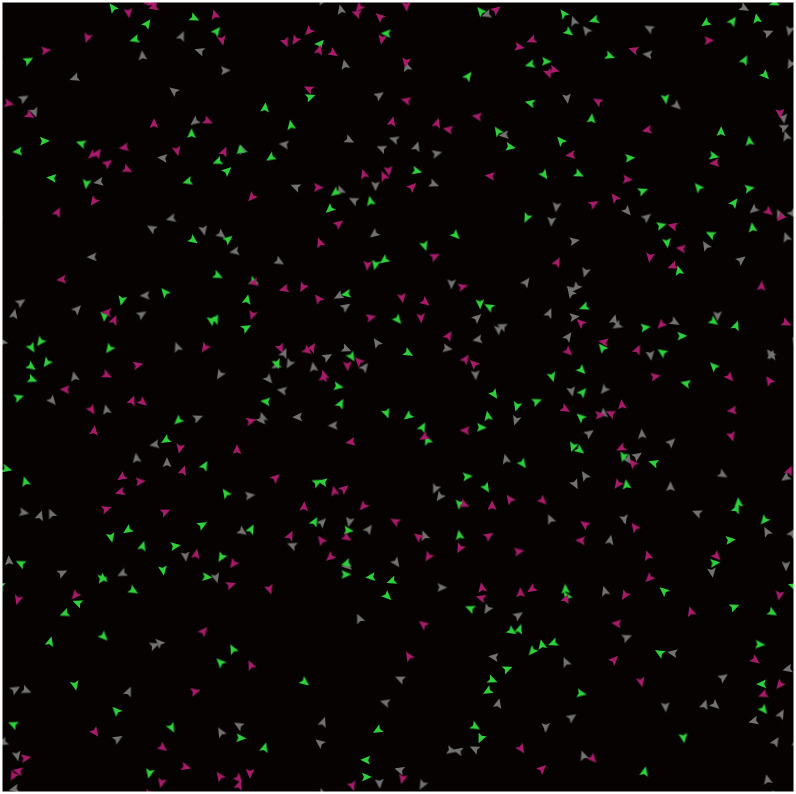
The experimental environment: Triangles represent the agents spread at different locations of the environment; different agent colors express different followed strategies.

To experiment the effectiveness of the proposed strategy, and compare it with other typical strategies, we have programmed a NetLogo version of the Axelrod prisoner dilemma tournament [30], enriched with spatial characteristics. Agents are placed in a planar spatial arena (wrapped as a torus), and continuously move in such arena (see Fig 1). Whenever an agent finds itself nearby another agent (i.e., in NetLogo terms, when they are in the same patch or in two adjacent patches), the two agents engage in a prisoner dilemma round, each playing its own strategy.

**Fig 2:**
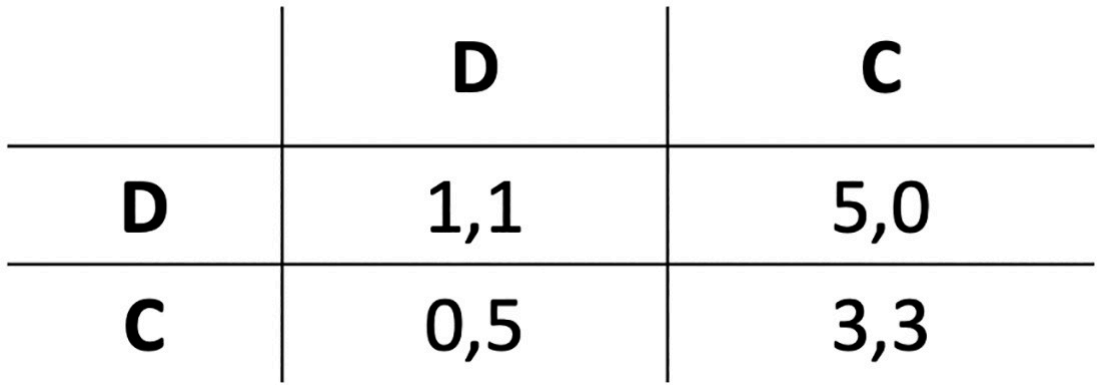
The adopted payoff matrix.

The specific payoff matrix adopted in the experiments (see Fig 2) considers *T* = 5, *R* = 3, *P* = 1, *S* = 0, but the presented results qualitatively hold independently of the specific values, provided that *T* > *R* > *P* > *S*.

When pheromones are not involved or not sensed by agents, the spatial nature of the model is irrelevant, and the tournament corresponds to a model of random encounters, in which agents randomly move in the environment and play the PD game when they meet another agent at the same location.

However, in the presence of agents that release pheromones in a specific portion of space that diffuse around, the spatial nature of the model becomes relevant, as there are regions of the arena that acquire specific properties.

Beside the classes of pheromone agents, the other classes of agents that we have implemented, and that can be instantiated in populations of variable dimension to compare with that of stigmergic agents, include:

- *random*: agents in this class randomly select (with equal probability) cooperation or defection. The behavior of random agents corresponds to the one of pheromone agents when they do not perceive an above-threshold concentration of pheromones.
- *defection*: agents in this class always select defect.
- *tit-for-tat*: agents in this class cooperate during their first encounter, and then reciprocate in the following ones (if the last met agents has defected, the tit-for-tat agent will defect as well in the next encounter, and vice versa).

We emphasize that we are not interested here in comparing the stigmergic strategy with other more sophisticated strategies (and in proving that it is the best of all) [31], but only in showing that it effectively promotes the emergence of cooperation.

### Qualitative analysis

**Fig 3:**
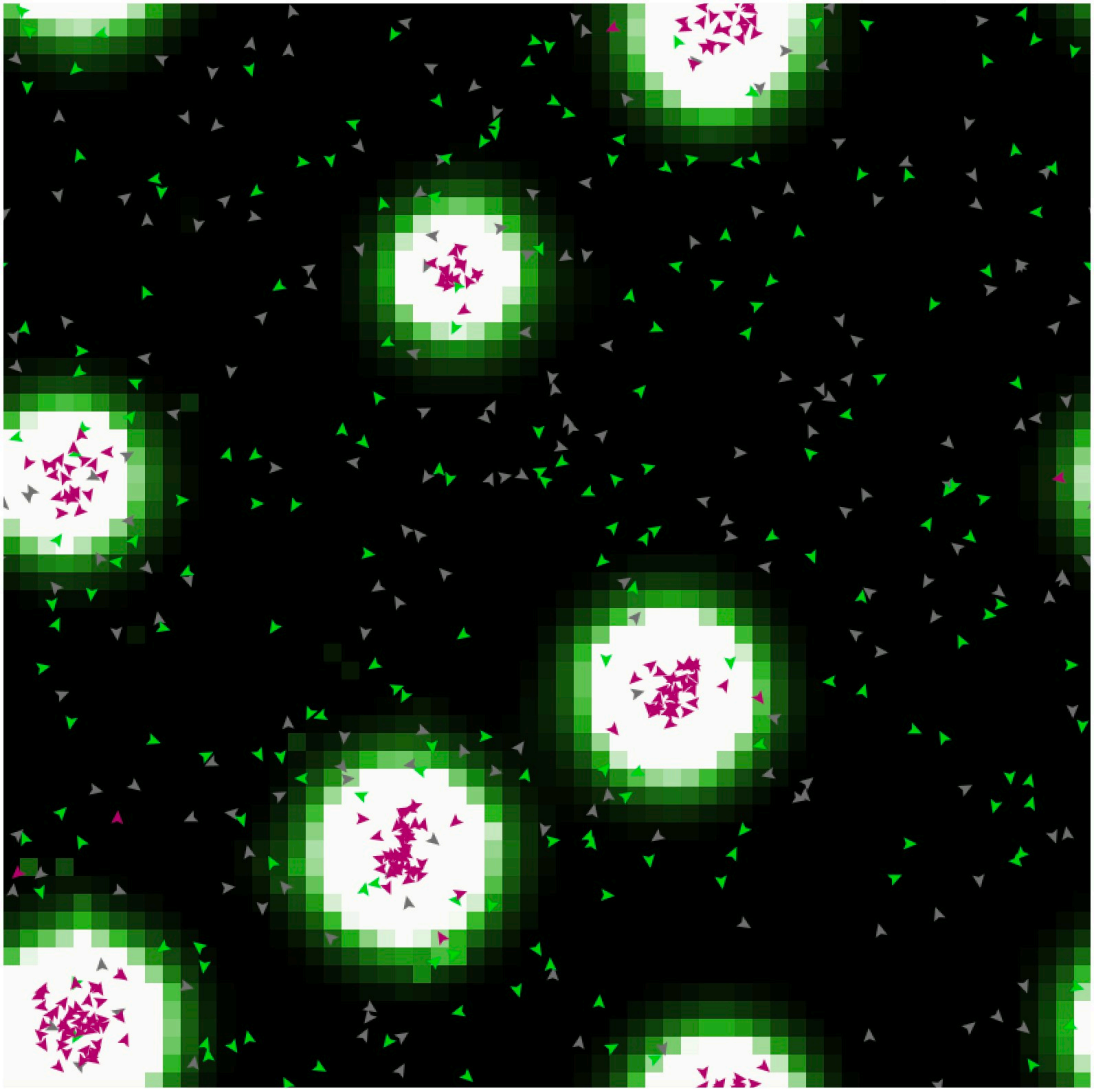
Typical patterns—“islands”—of cooperation emerging from the experiments.

Fig 3 shows the typical patterns of cooperation that tend to emerge in the experiments. The stigmergic agents (in red) emit pheromones around when they find cooperators, and tend to move in the direction of higher pheromones concentrations (pheromones are represented with shades of green gradually becoming white in regions with high concentrations). This leads to the formation of “islands” where cooperation is promoted and sustained. Agents following other strategies (represented by different colors depending on the strategy adopted), instead, are not sensitive to pheromones and keep moving randomly in the environment.

**Fig 4:**
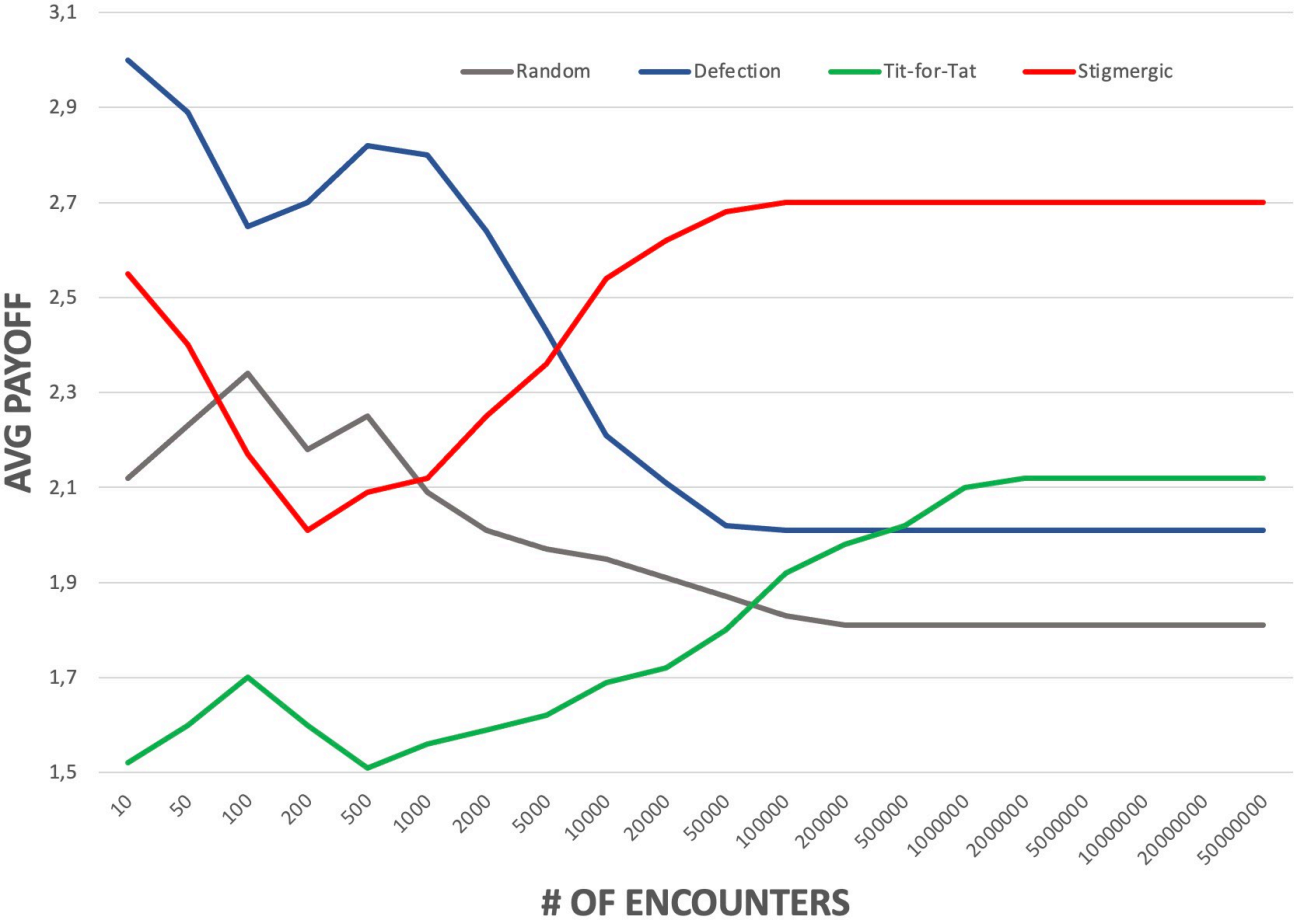
Typical evolution in time of the cumulative payoff for agents playing different strategies.

Fig 4 shows the typical evolution in time that has been observed in most of our experiments. The x-axis measures passing time in terms of the overall number of agent encounters. The y-axis measures the average payoff accumulated by agents playing different strategies.

At the beginning, stigmergic agents are not clustered and behave randomly, so their payoff is similar to the one of random agents, and penalized by the presence of defectors. The latter, in fact, exploits the random endeavor to cooperate of both random and stigmergic agents, and the initial cooperative endeavor of tit-for-tat agents. However, as soon as enough cooperative encounters between stigmergic agents take place, pheromones start diffusing in the environment, attracting other stigmergic agents, which makes stable islands of cooperation being formed. In such islands, cooperation takes the form of a sort of indirect reciprocity [7]: one agent *A* is akin to cooperate with another agents *B* not because it knows directly about *B*’s endeavour, but because it knows that in the islands of cooperation it is worth to cooperate.

The effect of cooperation by stigmergic agents “living” in such islands is that they start accumulating higher payoffs, eventually and stably becoming the class with the most profitable strategy. Defector agents are damaged by the presence of such cooperative islands, finding less chances of exploitation elsewhere. Tit-for-tat agents, on the other hand, due to their reciprocal behavior, take advantage as well from the islands of cooperation, when they happen to find themselves there, thus increasing their cumulative payoff over time.

### Quantitative analysis

Let us analyze more in detail the behavior of the different strategies in different conditions, in particular the average payoff accumulated by agents playing different strategies. The data refers to the average of 5 tournaments each involving an overall of 5×10^6^ bilateral agent encounters (a time sufficient to reach a stable behavior). We emphasize that, for all experiments, the standard deviation of the analyzed data is always below 10%.

In the experiments, performed in a torus shaped arena of 91×91 patches, the population of *defectors*, *random* and *tit-for-tat* agents is set to 200, whereas the population of *stigmergic* agents varies from 20 to 200. Fig 6 compares a similar scenario but in the absence of the *defector* agents population. For both experiments: the evaporation rate *ϵ* is set to 2.5% (the pheromone intensity decreases by one fortieth at each simulation step); the diffusion rate *δ* is set to 20% (the 20% of the pheromone is distributed to the neighbours at each simulation step). The pheromone intensity threshold *T*_*i*_ is set to zero (i.e., stigmergic agents starts cooperating as soon as they sense some, even small, amount of pheromones.

When the population of *stigmergic* agents is very low, the scenario lacks the critical mass needed to form stable islands of cooperation. From time to time *stigmergic* agents release pheromones due to a satisfying cooperative encounter, but the frequency of such events is too low, and the released pheromones evaporate before other *stigmergic* agents can reach the zones where such encounters have occurred. Thus, their accumulated payoff tend to be very close to that of the agents playing different strategies.

However, as soon as a critical mass of *stigmergic* agents is present in the environment, a phase transition occurs enabling stable islands of cooperation to emerge, which start attracting an increasing number of *stigmergic* agents. They can then take advantages of such islands by engaging in a large number of cooperative encounters, thus accumulating overall a much higher payoff than different strategies.

The presence of island of cooperation does not affect at all the overall payoff of agents playing the *random* strategy. Instead, *defectors* and *tit-for-tat* agents can take advantage of the presence of a large enough population of *stigmergic* agents, i.e., of the emergence of islands of cooperation. In fact, islands of cooperation enable passing by *defector* agents to temporarily exploit the cooperative behaviour of *stigmergic* agents, and increase their payoff w.r.t. the case in which islands of cooperation do not emerge. Yet, their overall payoff remains lower than that of stigmergic agents. Similar considerations apply to *tit-for-tat*, which can exploit islands of cooperation to successfully reciprocate cooperative behavior, but again with a cumulated payoffs notably lower than that of *stigmergic* agents. The same considerations for *stigmerigic*, *random* and *tit-for-tat* agents apply in the case *defector* agents are absent (see Fig 6).

**Fig 5:**
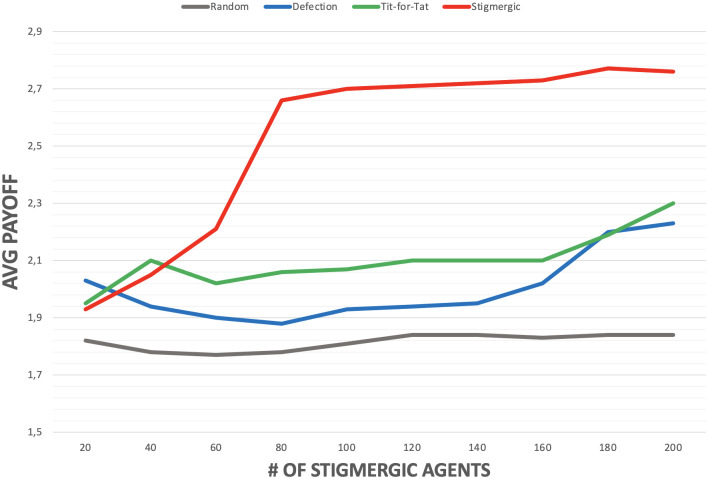
Average cumulative payoff, depending on the number of stigmergic agents (all other agents have a population of 200 each).

**Fig 6:**
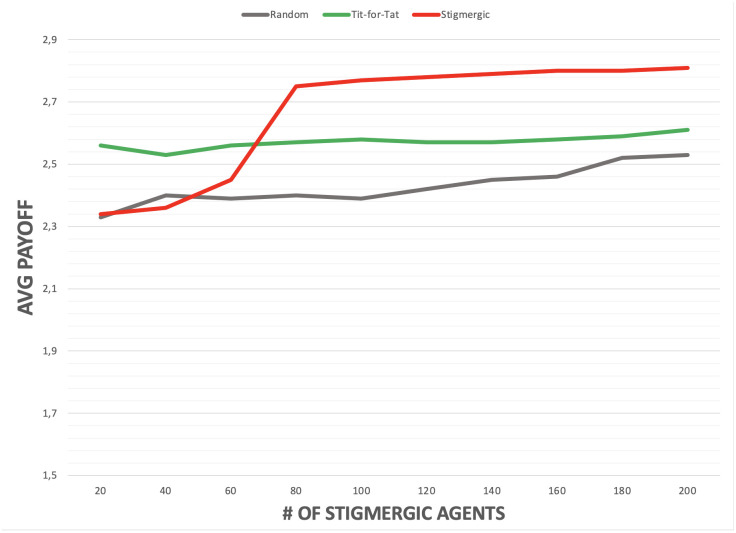
Average cumulative payoff, depending on the number of stigmergic agents, and without defector agents (all other agents have a population of 200 each).

**Fig 7:**
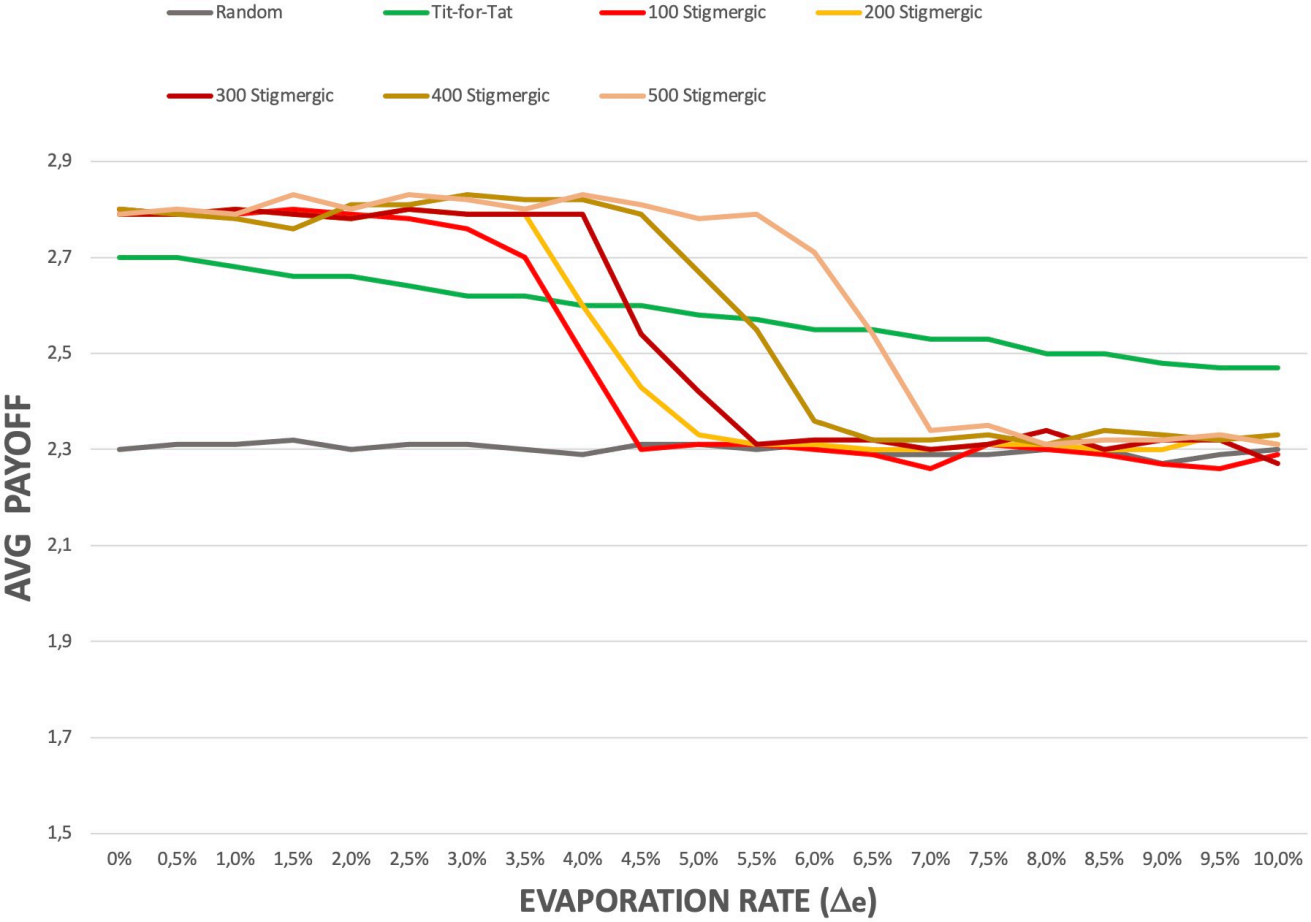
Effect of pheromone evaporation rate on the payoff of stigmergic agents.

Let us now analyse the impact of the evaporation rate. Figs 5 and 6 refers to experiments in which the evaporation rate of pheromones were set to 2.5%. However, it is worth analyzing the impact of such rate on the emergence of cooperation. Fig 7 shows what happens by varying the evaporation rate, while keeping the diffusion rate at 20%. In general, if the evaporation rate is too high, islands of cooperation do not emerge, and the payoff of *stigmergic* agents is comparable to that of *random* ones. This is due to the fact that pheromones emitted by agents in a region tend to vanish before other agents can reach that region to sustain the pheromone field. In addition, the figure shows that the more *stigmergic* agents live in the environment, the higher the evaporation rate that can be tolerated. In any case, a clear state transition is observed as soon as the evaporation rate exceeds a certain threshold, and islands of cooperation suddenly fail to emerge.

**Fig 8:**
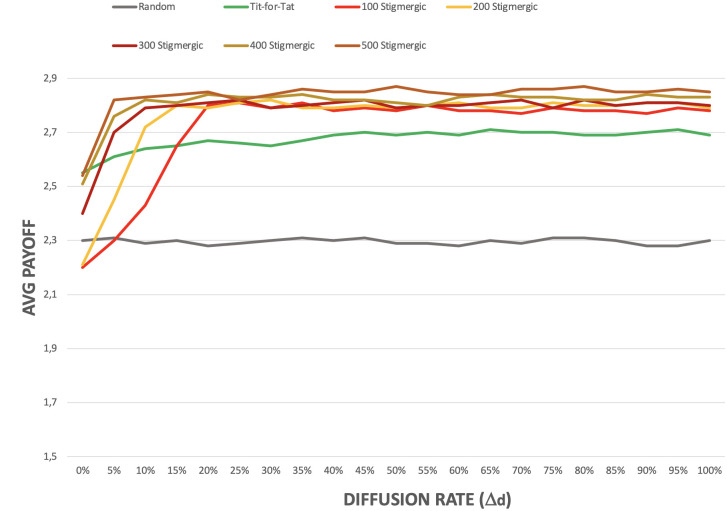
Effect of pheromone diffusion rate on the payoff of stigmergic agents.

Concerning the diffusion rate, instead, its impact on the overall behavior of the system is less relevant. Fig 8 shows the effect of different diffusion percentages with a fixed evaporation rate of 2.5%. In the absence of diffusion, pheromones remains allocated only in the location in which they were originally deposited. Thus, islands of cooperation fail to emerge, and in the best case (especially in the presence of a large number of stigmergic agents) only some very small islands emerge here and there. However, as soon as even a minimal amount of diffusion exists, islands of cooperation start to emerge and self-sustain, as shown by the growth of the payoff of stigmergic agents.

A peculiar—qualitative—effect of having very low diffusion rates is that islands of cooperation tend to assume “snake-like” shapes rather than uniform circular ones. This is due to the fact that the distribution of pheromones in the environment is mostly guided by the correlated random walk of agents rather than by the isotropic diffusion of pheromones.

### Impact of other parameters

The other parameters of our model have a less significant impact on the overall behavior of the system.

The intensity threshold *T*_*i*_ over which stigmergic agents start to cooperate, which was set to zero in our experiments, has only a short transitory impact on their behavior. In fact, once an agent starts perceiving and emitting pheromones, and consequently starts moving towards the region of higher pheromones concentration, it will always eventually reach a location in which the intensity of pheromones exceeds *T*_*i*_. Thus, a high *T*_*i*_ has the only effect of slightly delaying the moment at which an agent starts to cooperate).

The amplitude $\left[-\bar{\theta },\bar{\theta }\right]$ of the wiggle angle $\bar{\theta }$ that determines a random variation of the current direction of an agent has no significant effect on the behavior of the systems. In fact, any amplitude greater than zero and less than *π*/2 produces a correlated random walk enabling agents to eventually explore the whole environment. In the case $\bar{\theta }=0$ agents move deterministically always in the same direction. Nevertheless, the overall randomness of the directions in the populations let islands of cooperation emerge in any case. The only negligible side effect is that some stigmergic agents may fail in ever approaching an island of cooperation and join it.

The amplitude of the sniffle angle determines the cone of visibility for pheromones. For islands of cooperation to emerge, such angle must be over the threshold (around $\frac{\pi }{7}$) which enables an agent to sense pheromones in the 3 patches ahead of its current direction. Once it is over such threshold, the actual amplitude does not impact the overall behavior, apart from the qualitative aspect that sniff angle values close to the threshold make islands of cooperation tend to assume snake-like shapes (and for the same reasons this happens for low diffusion rates).

Finally, we would like to emphasize that the size of the arena is mostly irrelevant. What matters is the density of the agents populating the arena. Thus, all presented results holds for any arena dimensions, provided that the number of agents is scaled accordingly.

## Discussion

The above analysis can be summarized as follows:

- Pheromones represents a sort of externalized reputation measure, expressing the existence of spatial regions in which individuals with a cooperative endeavor can be found and indirectly reciprocated;
- Given the presence in the environment of a sufficient number of “stigmergic” individuals able to strategically exploit pheromones—i.e., moving by following pheromone trails and depositing pheromones when finding cooperative partners—spatial “islands of cooperation” emerge, that is spatial regions in which cooperative behavior can be effectively sustained;
- As a consequence of such cooperation, and as quantitatively shown by experiments, stigmergic agents can overall get higher payoffs than agents following different—non-stigmergic—strategies;
- The reported behavior is very robust with respect to most of the parameters of the model.

## Conclusions

In this paper we have presented a stigmergic—pheromone-based—mechanisms to let cooperation emerge in a society of self-interested agents. The basic motivation of the approach is to verify whether pheromones could take the role of externalized reputation, i.e., a reputation measure associated to regions of the environment rather that to individual agents.

The experiments performed in a tournament with repeated interactions have shown that pheromones, by letting agents perceive which regions of the environment are favourable to host cooperative endeavors, are indeed nudging agents to cooperate with the other agents in that regions. That is, pheromones induce indirect reciprocity.

Overall, the reported experiments corroborate the observation made in different fields (e.g., biology [20, 21], sociology [32], artificial intelligence and robotics [33, 34]) on the important role of stigmergic, i.e., indirect environment-mediated, interactions. When individuals of a system can affect the properties of the environment and are in turn affected by such properties—global self-organized cooperative behavior can arise and self-sustain. However, we also understand that the generality of our results are somewhat limited by the lack of a proper comparison with assessed reputation-based approaches and by the simplicity of our stigmergic model.

To overcome such limitations, in the future we intend to: *(i)* more deeply investigate the potential relations between reputation-based mechanisms and stigmergic ones, and evaluate whether and how the two mechanisms can co-exist and be harmonized [13]; *(ii)* couple the mechanism of attractive pheromones with a mechanism of repulsive ones, intended as a sort of punitive stigmergic mechanism to let agents effectively escape regions where defection is dominant [35]; *(iii)* verify if it is possible for agents to dynamically learn (or evolve) to exploit the pheromone-based mechanism [36].

## Supporting information

S1 File NetLogo simulation code. The code of the program used to perform the simulation and to extract the presented results. Requires installing the NetLogo simulation environment (https://ccl.northwestern.edu/netlogo).
(NLOGO)

S2 File Raw data in Excel. The data extracted from the NetLogo simulation code and analysed in the manuscript.
(XLSX)
